# Ionic liquid-iontophoresis mediates transdermal delivery of sparingly soluble drugs

**DOI:** 10.1080/10717544.2025.2489730

**Published:** 2025-04-21

**Authors:** Wenyan Gao, Wenmin Xing, Zhan Tang, Qiao Wang, Wenying Yu, Qi Zhang

**Affiliations:** ^a^ School of Pharmacy, Hangzhou Medical College, Hangzhou, China; ^b^ Key Laboratory of Neuropsychiatric Drug Research of Zhejiang Province, Hangzhou Medical College, Hangzhou, China; ^c^ Zhejiang Key Laboratory of Geriatrics and Geriatrics Institute of Zhejiang Province, Zhejiang Hospital, Hangzhou, China; ^d^ Institute of Library, Hangzhou Medical College, Hangzhou, China

**Keywords:** Ionic liquids, iontophoresis, transdermal delivery, sparingly soluble, permeation enhancer

## Abstract

Low solubility restricted transdermal penetration of drugs. We aimed to develop a novel ionic liquid-iontophoresis (IL-IS) technology and assess their efficacy and primary factors in facilitating transdermal drug delivery. Five choline-based ILs with different chain length were synthesized and validated, and the impact of IL and/or IS technology on transdermal penetration of model drugs were investigated. The results indicated that five groups of ILs synthesized in this study exhibited minimal level of toxicity, and the longer the chain of acid ligands of ILs, the greater the cytotoxicity. The longer chain of acid ligand was demonstrated superior solubilizing capabilities compared to the shorter chain. Cinnamic acid-choline-based IL ([Cho] [Cin]) significantly improved permeation of all three model drugs, and permeation quantity was linearly positively associated with the concentration of ILs. The 10 h cumulative permeation of aripiprazole applied with ILs alone was enhanced by about 14-fold when paired with IS, and the penetration was linearly positively associated with the concentration and current strength of the ILs. *In vivo* results indicated that IL and/or IS technology primarily facilitated drug penetration into the skin, with potential involvement of endocytosis in this process. This study demonstrated that [Cho] [Cin] exhibited a significant enhancement in the transdermal delivery of three sparingly soluble drugs. It further enhanced the transdermal permeation of weak base drug following with the combining IL and IS technology. These findings highlighted that the IL-IS technology holded promise for facilitating the transdermal delivery of sparingly soluble and weak base drugs.

## Graphical abstract

**Figure:**
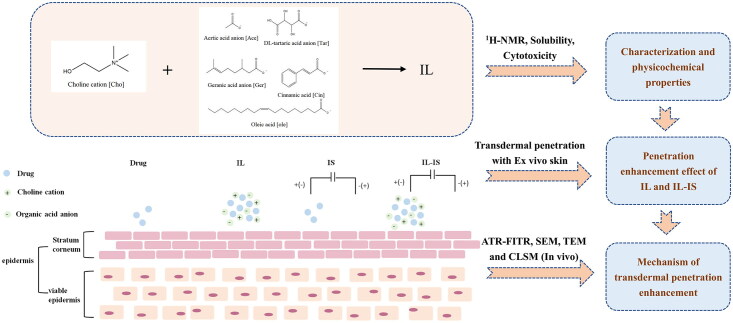

## 1. Introduction

The transdermal delivery system refers to a pharmaceutical technology wherein drugs permeate through various layers of the skin to provide therapeutic effects either locally or systemically. This technology has garnered significant interest in clinical applications due to its convenient application, ability to maintain a steady blood drug concentration, and minimal systemic toxicity and adverse effects (Prausnitz and Langer 2008, Wiedersberg and Guy 2014). However, it also has many shortcomings that can’t be ignored. The stratum corneum (SC), which constitutes the outermost layer of the skin, often serves as the primary barrier preventing the transdermal delivery of drugs (Phatale *et al.* 2022). The compact and intricate arrangement structure imposes restrictions on the permeation of exogenous chemicals. Hence, to improve the transdermal delivery of poorly soluble drugs, it is crucial to meet two simultaneous requirements: (1) increasing the drug’s solubility and (2) enhancing the drug diffusion coefficient. The solubility of the drug not only limits the rates at which it releases from a transdermal delivery system but also has a direct influence on the rate at which it diffuses through the skin (Lipinski *et al.* 2001; Alany 2017).

Over the past decade, there has been a rising interest in the application of ionic liquids (ILs) in the field of transdermal drug delivery systems. ILs are known for their liquid state at room temperature and consist of organic cations paired with either organic or inorganic anions. The anions and cations of ILs display significant size asymmetry and are distinguished by their substantial size. This, coupled with significant spatial site resistance, disrupts the ordered crystal structure of ILs, leading to their liquid-like properties (Correia *et al.* 2021; Ali *et al.* 2022). ILs has exceptional solubility characteristics for sparingly soluble drugs, hydrophilic drugs, and proteins, hence facilitating their transdermal penetration. For example, Samir Mitragotri synthesized a range of choline-based ILs, which had the ability to enhance the transdermal permeation of the insoluble drug Ruxolitinib. Additionally, the transdermal penetration effect was inversely correlated with the intermolecular forces between the components involved (Tanner *et al.* 2019).

Despite ILs growing utilization in the field of pharmaceutical formulations, the degradability and toxicity of ILs has been a subject of controversy. Imidazolium-based ILs has been present in the environment and capable of acting as a potential trigger in the auto-immune liver disease primary biliary cholangitis (Probert *et al.* 2018). It was worth noting that ILs made from natural sources, such as organic acids, amino acids, or choline, have low solubility for drugs. And they are easily metabolized *in vivo* and have been determined to be safe, as indicated by previous studies (Hou *et al.* 2013; Gomes *et al.* 2019; Dhattarwal and Kashyap 2021). Although IL can effectively improve the solubility of insoluble drugs, its ability to promote transdermal penetration is limited, making it difficult to achieve local or systemic effective therapeutic doses.

IS is a biophysical penetration method that involves the application of a low electrical current externally to facilitate ions across through the skin and into local tissues or systemic circulation (Batheja *et al.* 2006). This approach has the potential to decrease the dosage, mitigate adverse effects, and optimize therapeutic efficacy of drug. Our previous studies had also demonstrated that IS had a strong permeation-enhancing effect on the ionic drug terazosin (Jiang *et al.* 2021). As both ILs and IS are related to ion dissociation and migration, the unique characteristics of ILs make it perfectly compatible with IS technology. IS has a remarkable effect on the transdermal promotion of drugs, while ILs has been identified as agent capable of improving the solubility and conductivity of drugs that exhibit poor solubility. Can the issue of insufficient transdermal therapeutic dosage of lipophilic drugs be addressed through the integration of the solvent properties of IL and the iontophoretic effect of IS?

Hence, this study attempts to integrate the solvent characteristics of IL with the iontophoretic effect of IS to facilitate the transdermal delivery of sparingly soluble drugs. The primary factors and mechanism of IL-IS technology in transdermal delivery were also investigated. Three sparingly soluble drugs: apremilast, aripiprazole, and indomethacin were chosen as model drugs, choline-based ILs with five different acid ligands was designed and synthesized, and its physicochemical properties were characterized. The influence of drug properties, ILs composition, and current on the IL-IS technology for promoting transdermal drug delivery was conducted using excised rat skin *in vitro*, and the findings were further validated using *in vivo* penetration studies on rats. The mechanism by which IL-IS technology enhancing permeability was elucidated by the utilization of various analytical technologies, including infrared spectroscopy, fluorescence imaging. This study was firstly designed to combine ILs and IS technology for the transdermal delivery of poorly soluble drugs, which overcame the limitations of IS technology in the transdermal permeation of poorly soluble drugs by enhancing the solubilizing and conductive properties of ILs.

## 2. Materials and methods

### 2.1. Materials and animals

Apremilast, aripiprazole, indomethacin, and choline hydroxide were purchased from Sigma-Aldrich (St. Louis, Missouri). Acetic, DL-tartaric, cinnamic, geranic, and oleic acids were purchased from Aladin (Shanghai, China). All other chemicals were at least analytical grade. Sprague-Dawley rats animal model was necessary for pharmacodynamic evaluation *in vivo* in this experiment. Healthy female Sprague-Dawley rats (2 months, 200 ± 50 g) used in the experiments were obtained from Zhejiang Province Laboratory Animal Center (Hangzhou, China, certificate No: SCXK-(Zhe) 2019-0002). Before experimentation, the animals were treated humanely at room temperature (26 ± 2 °C). The authors had adhered to the ARRIVE guidelines. All animal experiments were performed in accordance with the animal care and use ethics. The animal experiment in this study had been approved by the Animal Care and Ethics Committee of Hangzhou Medical College (2022-008). At the end of the animal experiment, the rats were killed by dislocating. The human immortalized epidermal HaCaT cell was purchased from the iCell biotechnology company (Shanghai, China, catalog iCell-h066).

### 2.2. ILs design, synthesis, and measurement

**Figure 1.**
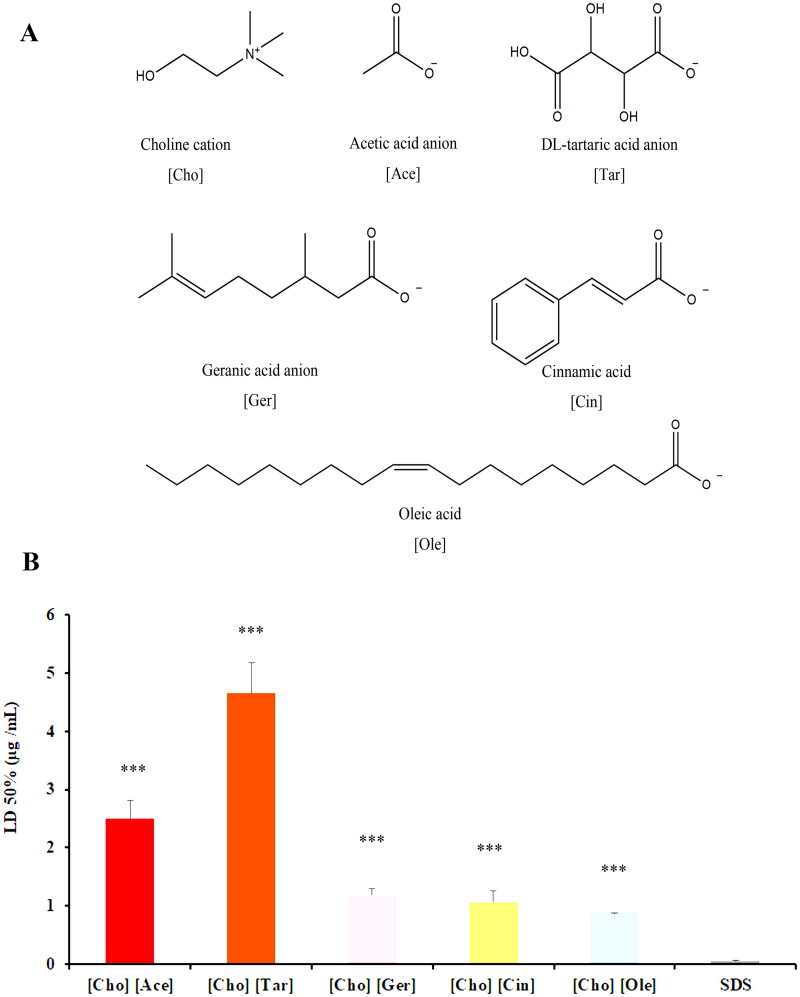
(A) Structure of choline cation and organic acid anion; (B) *in vitro* cytotoxicity of the ILs with regard to HaCaT cells. Data are presented as the mean ± SD (*n* = 3). Statistically significant differences between IL group and SDS group are shown by the asterisks (***p < 0.001).

To synthesize the ILs, representative compounds, such as a common short chain fatty acid (acetate acid), a binary organic acid (DL-tartaric acid), two medium chain fatty acids (geranic acid, cinnamic acid), and a long chain fatty acid (oleic acid) were selected (Figure 1(A)). ILs was prepared based on a previously reported procedure with minor modifications (Dharamdasani *et al.* 2020). Briefly, acid was allowed to react with choline hydroxide (44 wt% solution). The molar ratio of [Cho]: anion ([Ace], [Ger], [Cin], and [Ole]) was maintained at 1:1, and the molar ratio of [Cho]: [Tar] was maintained at 2: 1. The reaction mixture was stirred gently at 40 °C for 24 h. Thereafter, all the obtained samples were pre-frozen for 24 h in an ultralow-temperature refrigerator at −80 °C, followed by freeze-drying at −50 °C and −0.01 MPa for 72 h. This process eventually resulted in the freeze-dried ILs: [Cho] [Ace], [Cho] [Tar], [Cho] [Ger], [Cho] [Cin], and [Cho] [Ole].

And then, ^1^H-NMR spectra was obtained using a Bruker AVANCE NEO 500 instrument. ILs were dissolved in deuterochloroform and transferred to an NMR tube. Tetramethylsilane was chosen as an external standard, and chemical shifts were collected. Meanwhile, after diluted ILs to 20% (w/w) with water, a pH meter (PHS-3C, INESA, China) was used to measure the pH value, and electrical conductivity was measured with electroconductivity meter (DDS-307, CSDIHO, China). Viscosity was determined using a viscometer (DV2T, Brookfield, America).

### 2.3. Cytotoxicity

The cytotoxicity of ILs to HaCaT cells was assessed using the Cell Counting Kit 8 (CCK-8) assay. The cell suspension (100 µL) was transferred to a 96-well plate at 8 × 10^3^ cells/mL. Then, the 96-well plate was placed in a cell incubator. After 24 h, the medium was removed, and the cells were washed with phosphate-buffered saline (PBS, pH 7.4, 0.1 M). Then, ILs at different concentrations was added to each well for stimulation. After incubation for 24 h, CCK-8 solution (10 µL) was added to each well, followed by incubation for 2 h. The absorbance was measured at 450 nm using a microplate reader (SpectraMax iD5, Molecular Devices, America), and the cell viability was calculated.

### 2.4. Solubility

**Figure 2.**
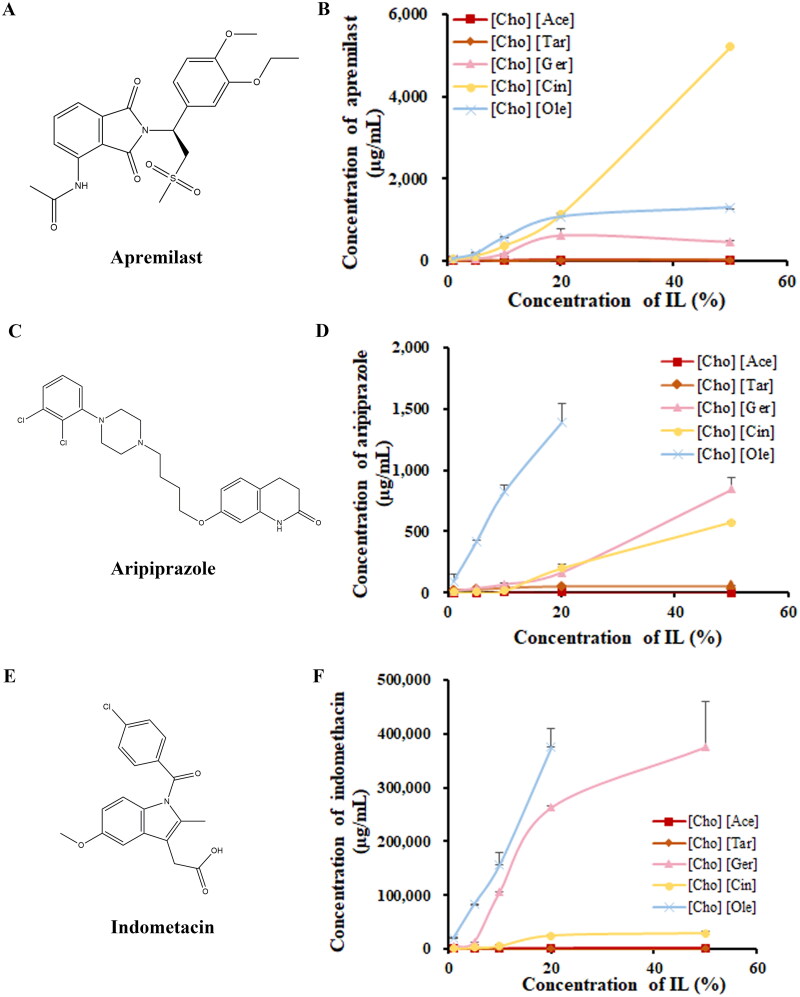
Structure and solubilities of model drug in different concentrations of ILs; (A,B) apremilast; (C,D) aripiprazole; (E,F) indomethacin. Data are presented as the mean ± SD (*n* = 3).

The flask shaking method was employed to evaluate the solubility of three model drugs [apremilast (neutral), aripiprazole (weakly basic), and indomethacin (weakly acidic)] in choline-based ILs with varying ligands and concentrations. The molecular structure of each drug is depicted in Figures 2(A,C,E). Except for [Cho] [Ole], the ILs was diluted with water to achieve a concentration ranging from 1 to 50%. Due to the semi-solid state of [Cho][Ole] at high concentrations, it was exclusively made at concentrations not exceeding 20%. The solubility of apremilast, aripiprazole, and indomethacin were determinated in deionized water, 1% sodium dodecyl sulfate (SDS), 0.01 M PBS (pH 4.5, 6.8, and 9.0) and different ligand, different concentrations of ILs, respectively, using HPLC. All measurements were conducted in triplicate.

The HPLC method with slight modifications was employed to detect the presence of apremilast, aripiprazole, and indomethacin. The analysis was conducted using the Agilent 1290 Series equipped with an autosampler and an ultraviolet detector. Separation was achieved on a Diamonsil-C18 column (4.6 × 150 mm, 5 μm; Dikma, China) at a column temperature of 30 °C and a flow rate of 1 mL/min. Three drugs were subjected to elution using a solvent mixture comprising trifluoroacetic acid (0.05%v/v) and acetonitrile, employing various solvent compositions and measure wavelengths. The elution of apremilast, aripiprazole, and indomethacin occurred at solvent ratios of 48:52, 42:58, and 52:48, respectively, while the corresponding measure wavelengths were 230, 254, and 320 nm.

### 2.5. Transdermal penetration

The transdermal penetration of ILs and IL-IS was investigated by skin permeation experiment using multi-functional transdermal diffusion instrument and horizontal diffusion cells at 32 ± 0.5 °C, based on a previously report (Niu *et al.* 2019). Anesthetize rats by intraperitoneal injection with a dose of 1.0 g/kg urethane solution. Then remove the back hair using a pet shaver. Carefully remove the muscle tissue from the inner side of the skin with a surgical knife, cut it into small pieces, soak it in physiological saline for 30 min, cover the diffusion cell opening, and secure it with a clip. Intact rat skin was positioned between two cells, with the SC facing the donor compartment. The drugs were precisely measured at a weight of 4.00 mg each and subsequently dissolved in 4 mL of ILs or other control solutions, and added in donor compartment. The 0.01 M PBS (pH 6.8, 4.0 mL) was chosen as receptor medium. Sampling of ILs was conducted at various time points, specifically at 0, 4, 6, 8, 10, and 24 h. Following each sampling, 2.0 mL (all receptor medium was removed at the 10-h time point) of receptor medium was replaced with fresh PBS solution. The transdermal penetration of IS or IL-IS was conducted using an electroosmosis apparatus (Dajia DL-Z, China) to ensure a consistent current intensity of 0–0.5 mA. The direction of the current flow from the donor compartment to the acceptor compartment was defined to be positive current, while the reverse direction exhibited a negative current in this study. A receiving solution (2.0 mL) was collected at 1, 2, 4, 6, 8, and 10 h. After skin permeation experiments, rat skins were subsequently removed from cells, cut into pieces, and immersed in methanol for 12 h. The samples were then diluted with mobile phase at a ratio of 1:10. Following centrifugation for 10 min at 9000 rpm, the resulting supernatants were analyzed using HPLC, as previously described.

The drug skin permeation amount of solution system was calculated through concentration and volume of each sampling point:

$$Q=[C_{n}\times V_{o}+\sum_{i=1}^{n-1}C_{i}\times V]/S$$

Among them, *Q* (μg/cm^2^): per unit area of cumulative penetration after several hours; *C*: drug concentration; *V*: volume of the receiver solution at each time; *S*: effective penetration area. In this experiment, *V_o_*: 4.0 mL; *V*: 2.0 mL (24 h point: 4 mL); *S*: 0.74 cm^2^.

The drug skin retention amount was calculated as follows:

R=M/S

Where *R* was drug skin retention amount (μg/cm^2^), *M* was drug amount in skin (μg), and *S* was effective penetration area of skin (0.74 cm^2^).

### 2.6. Mechanism of transdermal penetration enhancement

#### 2.6.1. Attenuated total reflection Fourier infrared spectrum (ATR-FITR)

ATR-FTIR study was used to investigate the effecting on SC of IL and/or IS. The spectra were collected with an IR spectrometer (Nicolet iS 10, Thermo Scientific, USA). A control gel was prepared by adding 4 g of hydroxypropyl methylcellulose (HPMC) K4M to 100 mL of water. Similarly, a 20% [Cho] [Cin] gel was prepared by adding 4 g of HPMC K4M to 100 mL of 20% [Cho] [Cin]. Following a 12-h fasting period, the hair on the backs of female rats was cleanly shaved after anesthesia with a dose of 1.0 g/kg urethane. The rats were randomly separated into four groups and were treated as follows:(1) control gel; (2) IL group: a 20% [Cho] [Cin]] gel; (3) IS group: a control gel with negative current at a current intensity of 0.3 mA; (4) IL-IS group: a 20% [Cho] [Cin] gel with negative current at a current intensity of 0.3 mA. The skins were cut for test 10 h later, and washed with PBS. Then, the skin samples were fixed on the infrared spectrometer, and the SC was used as the detection surface. The spectra were collected in the region of 4000–400 cm^−1^.

#### 2.6.2. Scanning electron microscope (SEM) and transmission electron microscope (TEM)

SEM enables the acquisition of high-resolution images with a three-dimensional appearance, allowing for the detailed visualization of intricate surface features of the skin. The skin samples used in this experiment were prepared according to the procedure described in Section 2.6.1, with minor modifications. Briefly, the samples were initially cut into 1 cm^2^ pieces. They were then fixed in a 2.5% glutaraldehyde solution at 2–4 °C overnight. Following fixation, the tissues underwent dehydration, drying, and were subsequently coated with a gold-palladium alloy. The samples were then examined using a Scanning Electron Microscope (JSM-IT200, JEOL, Japan).

TEM is widely regarded as the most efficacious technology for visualizing the intricate ultrastructural features of the stratum corneum (SC). The images presented in this study depict the ultrastructure of the SC following various treatments. The skin samples used in this experiment were prepared according to the procedure described in Section 2.6.1, with minor modifications. Briefly, after the 1 cm^2^ tissue block was rinsed, dehydrated, and infiltrated, the specimen was encapsulated in capsules containing an embedding medium. The specimen was then subjected to a temperature of 70 °C for ∼9 h. The resulting ultrathin sections were subsequently stained with uranyl acetate and alkaline lead citrate for 15 min each. The sections were then observed using a Transmission Electron Microscope (HT7700, HITACHI, Japan).

#### 2.6.3. Histological evaluation

Female rats were randomly divided into four groups: control, IL, IS, and IL-IS group. The hair on the dorsal region of was carefully removed, and the dorsal region of each rat was then exposed to the application of the prepared gel sample, and/or electroosmotic treatment as Section 2.6.1. The skin was removed after 6 h of daily treatment after 7 consecutive days. Subsequently, the treated skin sections were removed and fixed in 4% glutaraldehyde for 24 h. Then, the fixed skin was dehydrated by placing them sequentially into ethanol solutions of varying concentrations, starting from low to high: 70, 80, 90, 95, and 100%. Each ethanol concentration was used for an immersion period of 1 h. After dehydration, the skin was placed in xylene for clearing for 20 min. Then infiltrated with paraffin, embedded, sectioned, dewaxed, and rehydrated, followed by hematoxylin staining. Finally, the samples were dehydrated, cleared, and mounted. The histological analysis was carried out using a digital pathology scanning system (Pannoramic SCAN, 3D HISTECH, Hungary).

#### 2.6.4. Confocal laser scanning microscopy (CLSM)

CLSM (LSM710, Carl Zeiss, German) was used to visualize transdermal pathway of drug in skin. As indicated in transdermal penetration experiments, the utilization of IL-IS technology had the potential to enhance the transdermal penetration of alkaline drugs. Hence, Rhodamine B, an alkaline fluorescent probe, was chosen as the representative drug to investigate the *in vivo* penetration capacity and pathway of the drug. To investigate the occurrence of endocytosis during drug penetration, the study involved the utilization of specific endocytosis inhibitors, namely nystatin (chelating cholesterol to inhibit endocytosis), indomethacin (inhibiting cell membrane caveolin-mediated endocytosis) and chlorpromazine (inhibiting clathrin-mediated endocytosis). rhodamine B was chosen as fluorescein, which structure was water-insoluble and weakly basic. An appropriate amount of rhodamine B was weighed and added to the prepared control gel or 20% IL gel to achieve fluorescent gel. Endocytic inhibitors were prepared as follows: 926.11 µg/mL of nystatin, 357.79 µg/mL of indomethacin, and 6 µg/mL of chlorpromazine in 0.5% DMSO solution. Female rats were subjected to a 12-h fasting period and subsequently administered a dose of 1.0 g/kg urethane to anesthesia. The rats’ back hair was completely shaved before experimentation. Following a 1-h application of the inhibitor, the skin was coated with the prepared gel and/or subjected to electroosmotic treatment for a duration of 10 h. Subsequently, the skin was removed and washed with PBS. The skin samples were then embedded and sectioned at −20 °C, resulting in 20 µm thick sections. CLSM was employed to observe the localization and depth of fluorescence retention.

### 2.7. Statistical analyses

The one-way ANOVA was used to evaluate the statistical differences. A *p*-value <0.05 was considered as statistically significant.

## 3. Results and discussion

### 3.1. Characterization and physicochemical properties of ILs

#### 3.1.1. Physicochemical properties

The ^1^H-NMR characteristic peaks of the five groups of ILs were shown in Table S1, and the characteristic peaks were consistent with the structure of the assumed ILs. The general properties of ILs were shown in Table 1. The pH values of [Cho] [Ace] and [Cho] [Cin] were 9.37 and 8.27, respectively, indicating a weakly basic character. The pH values of [Cho] [Tar], [Cho] [Ger], and [Cho] [Ole] were very similar, around 6.5, suggesting a weakly acidic character. The viscosity was proportion to the chain length of the acid ligand. The observed trend in conductivity was as follows: [Cho] [Ace] > [Cho] [Tar] > [Cho] [Cin] > [Cho] [Ger] > [Cho] [Ole], which suggested a negative correlation between electrical conductivity and the chain length of the acid ligand.

**Table 1.** General properties of ILs.^a^

ILs	Ratio^b^	Appearance (room temperature)	20%IL		
			Viscosity (Pa·s)	Electric conductivity (ms/cm)	pH
[Cho] [Ace]	1:1	Transparent liquid	1.69 ± 0.08	32.12 ± 0.03	9.37 ± 0.01
[Cho] [Tar]	1:2	Transparent liquid	1.48 ± 0.01	28.74 ± 0.01	6.56 ± 0.05
[Cho] [Ger]	1:1	Light yellow liquid	1.95 ± 0.01	17.10 ± 0.01	6.53 ± 0.02
[Cho] [Cin]	1:1	Golden liquid	1.97 ± 0.04	18.60 ± 0.06	8.27 ± 0.01
[Cho] [Ole]	1:1	Yellow wax-like semisolid	7.63 ± 0.05	13.75 ± 0.01	6.55 ± 0.01

^a^ Data presented as the mean ± SD (*n* = 3).

^b^It was the molar ratio of ratio of acidic ligand and choline.

#### 3.1.2. Cytotoxicity

In this study, five groups of ILs were synthesized by using cationic choline-based and short-chain fatty acids (acetate), medium-chain fatty acids (geraniol, cinnamic acid), long-chain fatty acids (oleic acid), and binary organic acids (tartaric acid), which demonstrated a high level of safety. Figure 1(B) illustrated the median lethal dose (LD 50) of choline-based ILs with five different acid ligands. It was suggested that the cytotoxicity of ILs followed a descending order: [Cho] [Ole] > [Cho] [Cin] > [Cho] [Ger] > [Cho] [Ace] > [Cho] [Tar]. The cytotoxicity of the binary organic acid-based IL was found to be the lowest, indicating a higher level of safety. Additionally, the LD50 value of each IL was much higher than that of SDS, suggesting that ILs were significantly less hazardous to cells compared to SDS. The safety profile of the ILs consisting of short chain fatty acids was shown to be superior to that of ILs constituted of long chain fatty acids. The reason might be that long-chain acid ligands were more likely to interact with the phospholipids of cell membranes, affecting membrane stability (Probert *et al.* 2018, Liu A *et al.* 2024).

#### 3.1.3. Solubility

As shown in Table 2, the solubility of three model drugs, apremilast, aripiprazole, and indomethacin in water was found to be significantly limited, which were 21.8 ± 3.8, 0.94 ± 0.15, and 28.5 ± 0.7 μg/mL, respectively. In the positive control group, 1% SDS, the solubility increased significantly to 56.7, 844.9, and 470.4 times of that in water, respectively. The solubility of apremilast, aripiprazole, and indomethacin in different pH media was consistent with their pH characteristics.

**Table 2.** Solubilities of apremilast, aripiprazole, and indomethacin in different solvents.^a^

Solvents	Solubilities (µg/mL)		
	Apremilast	Aripiprazole	Indomethacin
20%[Cho] [Ace]	22.8 ± 2.5	3.7 ± 0.1	1306.2 ± 17.0
20%[Cho] [Tar]	21.8 ± 4.2	55.2 ± 0.9	200.5 ± 1.9
20%[Cho] [Ger]	638.7 ± 34.8	164.7 ± 64.6	262,637.0 ± 2446.5
20%[Cho] [Cin]	1123.0 ± 47.0	200.0 ± 17.1	25,253.5 ± 6599.9
20%[Cho] [Ole]	1080.9 ± 58.6	1387.3 ± 156.2	374,907.9 ± 35,033.1
Purified water	21.8 ± 3.8	0.94 ± 0.15	28.5 ± 0.7
pH 4.5	18.3 ± 7.2	151.7 ± 16.3	2629.9 ± 647.75
pH 6.8	13.5 ± 0.5	8.5 ± 3.2	21.5 ± 5.7
pH 9.0	6.8 ± 1.0	0.79 ± 0.5	2230.8 ± 136.1
1%SDS	1235.9 ± 59.2	794.2 ± 0.6	13,408.2 ± 1104.6

^a^ Data presented as the mean ± SD (*n* = 3).

The solubility of apremilast in 20% [Cho] [Ole] and [Cho] [Cin] displayed solubility capabilities similar to those of 1% SDS. Notably, the solubility of aripiprazole in 20% [Cho] [Ole] was much higher than that in 1% SDS. Furthermore, the solubility of indomethacin in 20% [Cho] [Ole], [Cho] [Cin], [Cho] [Ger], [Cho] [Ace], and [Cho] [Tar] increased to 13154.6, 886.1, 9215.3, 7.8, and 45.7 times, respectively, compared to its solubility in water, superior compared to that in 1% SDS. The findings of this study indicated that the incorporation of ionic liquids (ILs) resulted in a noteworthy improvement in the solubility of the three selected model drugs. It was observed that the solubility of ILs varied among the different drugs, depending on the specific acid ligands utilized. Specifically, the solubility of ILs containing medium and long chain acid ligands exhibited a significantly superior performance compared to ILs containing short chain acid ligands, for all three model drugs. Additionally, Figures 2(B,D,F) illustrated the equilibrium solubility of these drugs in ILs of varying concentrations. The solubilities of [Cho] [Ole], [Cho] [Cin], and [Cho] [Ger] indicated a positive relationship with concentration before reaching saturation. Moreover, these substances had notable solubilization effects. The solubility of [Cho] [Ace] and [Cho] [Tar] did not exhibit substantial changes as the concentration increased, suggesting the presence of mild solubilization effects. The solubility of [Cho] [Ger] for apremilast and indomethacin exhibited a saturation pattern as the concentration increased. The inclusion of medium and long chains in the structure might potentially offer a lipophilic terminus, which can effectively encapsulate lipophilic drugs and assume a more prominent role in enhancing solubilization (Arellano *et al.* 2025).

The effects of ILs on the solubility of different drugs were also influenced by the acidity or alkalinity of the drugs. For example, indomethacin was a weakly acidic drug with a pKa value of 4.5. When using ILs (such as [Cho][Cin] and [Cho][Ole]) composed of choline and weak organic acids, these ILs were weakly alkaline. The weakly basic ILs could interact with the carboxyl group of indomethacin, forming stable ion pairs or hydrogen bonds, thereby significantly enhancing its solubility. Therefore, ILs exhibited a stronger solubilizing effect on indomethacin compared to more neutral or weakly basic drugs, such as apremilast and aripiprazole.

### 3.2. Penetration enhancement effect of ILs

The primary influencing factors of ILs on the transdermal permeation/retention considered in this study were the drug, the type, and the concentration of the ILs (Furuishi *et al.* 2024). The impact of these factors on percutaneous permeability was investigated through *in vitro* skin permeation experiments.

#### 3.2.1. Properties of model drugs and types of ILs

**Figure 3.**
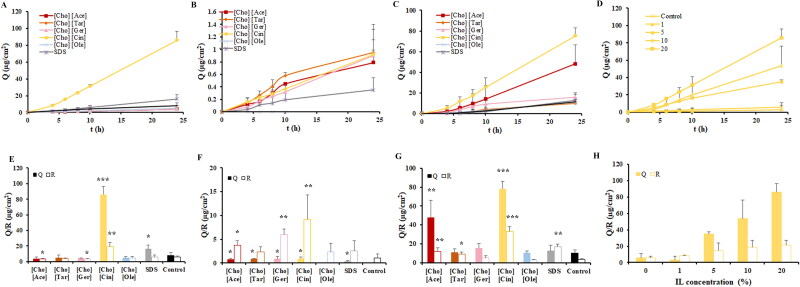
Effect of ILs type and concentration on the 24 h permeation/retention of model drugs when using IL alone. Data are presented as the mean ± SD (*n* = 4–6). Statistically significant differences of permeation/retention between IL group and control group are shown by the asterisks (*p < 0.05; **p < 0.01; ***p < 0.001). (A) Permeation profiles of apremilast under different IL types. (B) Permeation profiles of aripiprazole under different IL types. (C) Permeation profiles of indomethacin under different ILs types. (D) Permeation profiles of apremilast under different ILs concentrations. (E) the permeation/retention amounts of apremilast under different IL types. (F) The permeation/retention amounts of aripiprazole under different IL types. (G) The permeation/retention amounts of indomethacin under different IL types. (H) The permeation/retention amounts of apremilast under IL concentrations.

This section examined the impact of different types of ILs and model drugs on transdermal permeability, with the IL concentration set at 20%. The results of this investigation were depicted in Figure 3. Figures 3(A,E) were shown that the transdermal permeation/retention of apremilast was relatively low, with a cumulative permeation of just 8.1 µg/cm^2^ in water after 24 h, and a retention quantity of merely 6.1 µg/cm^2^. The [Cho] [Cin] exhibited the highest level of permeation/retention enhancement, as evidenced by a 24 h cumulative penetration that was 10.6-fold/3.1-fold greater than that of water, about 5.3-fold/3.1-fold greater than that of 1% SDS. [Cho] [Ace], [Cho] [Tar], [Cho] [Ole], and [Cho] [Ger] did not have significant permeation/retention enhancement effects. Figures 3(B,F) illustrated that the transdermal permeation/retention of aripiprazole was extremely low. In water, it exhibited negligible permeation ability, with only a trace amount retained in the skin, measuring 1.1 µg/cm^2^. However, when subjected to 1% SDS, the cumulative permeation after 24 h increased to 0.35 µg/cm^2^, and the retention amount rose to 2.6 µg/cm^2^. [Cho] [Ger], [Cho] [Ace], and [Cho] [Tar] demonstrated certain permeation enhancement effects on aripiprazole, but there was no significant difference observed between the different ILs. The cumulative permeation after 24 h ranged from ∼0.79 to 0.95 µg/cm^2^, with a retention amount of 2.4–6.0 µg/cm^2^. Compared to permeation, the improvement of skin retention of aripiprazole by ILs was more obvious. On the other hand, [Cho] [Cin] did not show a significant difference in cumulative permeation after 24 h, but its retention amount surpassed that of other ILs. Lastly, [Cho] [Ole] displayed weak permeation/retention ability for aripiprazole. Furthermore, Figures 3(C,G) illustrated that the transdermal permeation/retention of indomethacin in water was also low, and the cumulative permeability and retention of indomethacin at 24 h were only 10.06 µg/cm^2^ and 6.1 µg/cm^2^, respectively. The cumulative permeability of [Cho] [Cin] and [Cho] [Ace] at 24 h was 10.6 and 4.8 times of that of water, and the retention amount was 4.0 and 1.4 times of that of water. There was no significant effect of [Cho] [Tar], [Cho] [Ole], [Cho] [Ger], and 1%SDS on promoting permeation/retention. Overall, ILs with different acid ligands exhibited distinct permeation/retention enhancement properties on the model drugs. [Cho] [Cin] demonstrated notable permeation/retention enhancement effects on apremilast, aripiprazole, and indomethacin.

#### 3.2.2. Concentration of ILs

The investigation analyzed the influence of ILs concentration utilizing apremilast and [Cho] [Cin] as model drugs and types of ILs, respectively. The results of this investigation were depicted in Figures 3(D,H). Results showed that the transdermal permeation of apremilast increased with an increase of [Cho] [Cin] concentration, which indicated that there was a linear correlation between the concentration of [Cho] [Cin] and the transdermal permeability of apremilast within a certain concentration range. When the concentration of [Cho] [Cin] was 0–20%, there was no significant change in the skin retention amount.

### 3.3. Penetration enhancement effect of IL-IS

The primary influencing factors of IL-IS on the transdermal permeation/retention considered in this study were the drug, the type and concentration of ILs, and the direction and intensity of current, among others (Tijani *et al.* 2024). The impact of these factors on percutaneous permeability was investigated through *in vitro* skin permeation experiments with a consistent current intensity.

#### 3.3.1. Current direction

**Figure 4.**
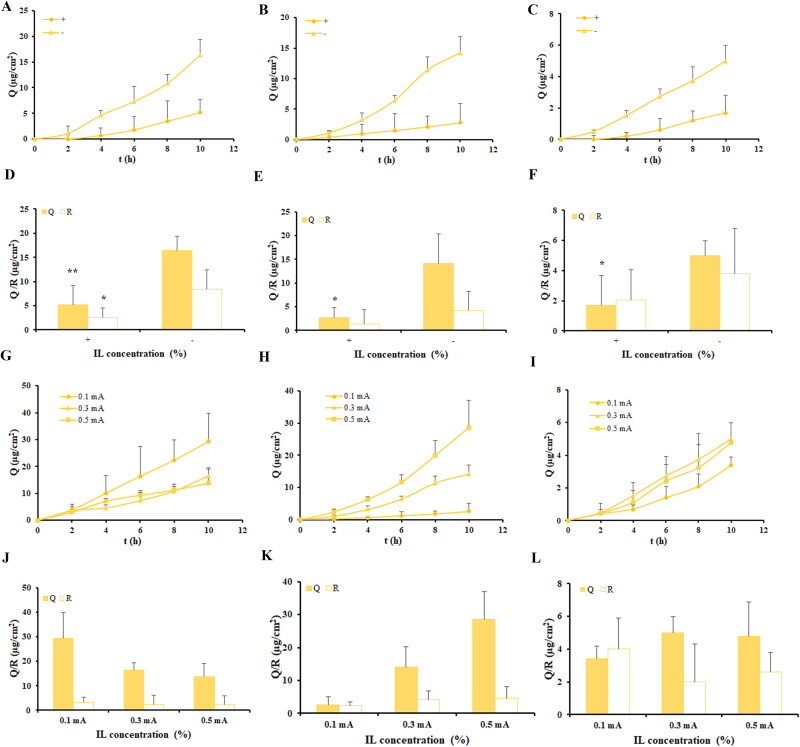
Effect of current direction on the 24 h permeation/retention and current intensity on the 10 h permeation/retention of model drugs when using IL-IS. Data are presented as the mean ± SD (*n* = 4–6). Statistically significant differences of permeation/retention between positive current group and negative current group are shown by the asterisks (*p < 0.05; **p < 0.01). (A) Permeation profiles of apremilast at different current directions. (B) Permeation profiles of aripiprazole at different current directions. (C) Permeation profiles of indomethacin at different current directions. (D) The permeation/retention amounts of apremilast at different current directions. (E) The permeation/retention amounts of aripiprazole at different current directions. (F) The permeation/retention amounts of indomethacin at different current directions. (G) Permeation profiles of apremilast at different current intensities. (H) Permeation profiles of aripiprazole at different current intensities. (I) Permeation profiles of indomethacin at different current intensities. (J) The permeation/retention amounts of apremilast at different current intensities. (K) The permeation/retention amounts of aripiprazole at different current intensities. (L) The permeation/retention amounts of indomethacin at different current intensities.

The influence of current direction on the enhancement of permeation/retention for IL-IS was assessed, with the concentration of [Cho] [Cin] and the intensity of the current set at 20% and 0.3 mA, respectively. As shown in Figures 4(A–F), the transdermal penetration effect of the three model drugs varied depending on the direction of the current. After 10 h of cumulative penetration, the permeation/retention of apremilast in the negative current was about 3.2-fold/3.4-fold greater than that in the positive current. The extent of aripiprazole permeation/retention at the negative electrode was ∼5.0-fold/3.0-fold higher compared to its permeation at the positive electrode. The indomethacin exhibited ∼3.0-fold/1.9-fold higher permeation/retention at the negative electrode compared to the positive electrode. It was indicated that the three model drugs exhibited enhanced permeation/retention when subjected to a negative current direction. Hence, a consistent choice was made to examine the effect on skin permeation/retention by focusing exclusively on the negative direction of current.

#### 3.3.2. Current intensity

Following the incorporation of IS technology based on 20% [Cho] [Cin], the effect of various current intensities (0.1–0.3 mA) in the negative current direction on the transdermal permeation/retention of three model drugs were investigated and the results were shown in Figures 4(G–L). The transdermal permeation of aripiprazole showed an increase in response to an elevation in current, there was a positive linear correlation between current intensity and the transdermal permeation of aripiprazole. However, the transdermal penetration of apremilast and indomethacin was not significantly enhanced with the increase in current intensity. In fact, it decreased to some extent, suggesting that current might inhibit the transdermal permeation of apremilast and indomethacin. Furthermore, changing the current intensity did not result in a significant difference in the retention amount of apremilast, aripiprazole, and indomethacin.

#### 3.3.3. Properties of model drugs

**Figure 5.**
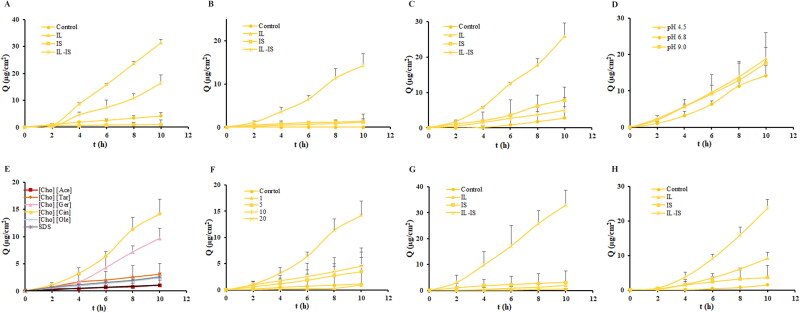
Other effects on the 10 h permeation of model drugs when using IL-IS. Data are presented as the mean ± SD (*n* = 6). (A) Permeation profiles of apremilast at different penetration enhancement strategies. (B) Permeation profiles of aripiprazole at different penetration enhancement strategies. (C) Permeation profiles of indomethacin at different penetration enhancement strategies. (D) Permeation profiles of aripiprazole at different pH values. (E) Permeation profiles of aripiprazole at different IL types. (F) Permeation profiles of aripiprazole at different IL concentrations. (G) Permeation profiles of butenafine at different penetration enhancement strategies. (H) Permeation profiles of terazosin at different penetration enhancement strategies.

This investigation explored the effects of model drugs on transdermal permeation, employing a [Cho] [Cin] concentration of 20% and a negative current of 0.3 mA. As shown in Figures 5(A–C), IL and IL-IS significantly increased the transdermal permeation of apremilast by 31-fold and 16-fold respectively, after 10 h of cumulative penetration. IS had little effect on the permeation of apremilast. For aripiprazole, only IL-IS showed a 14-fold increase in the permeation, while IL and IS had little effect on the permeation. For indomethacin, IL and IS showed a 3.3-fold and 1.6-fold increase in the permeation respectively, while IL-IS had little effect on the permeation. The permeation enhancing effects of the IL, IS, and IL-IS technologies varied among the three model drugs. Specifically, the effects of IL were more pronounced on apremilast and indomethacin, while the effects of IL-IS were more pronounced on aripiprazole. In combination with IS, the transdermal permeation of apremilast and indomethacin were constrained, suggesting that this technology exhibited selectivity toward drug’s physicochemical property (Touma *et al.* 2023). It was possible that both apremilast and indomethacin were influenced by the same ionic inhibition of acid ligands. Consequently, aripiprazole was selected as the model drug to investigate the influence of pH value and other IS-related factors, as shown in Figure 5(D), there was no significant change in the permeation of aripiprazole at pH 4.5, pH 6.8, and pH 9.0.

#### 3.3.4. Types and concentration of ILs

Figure 5(E) illustrated the impact of various ILs on the transdermal permeation, employing an IL concentration of 20% and a negative current intensity of 0.3 mA. The application of IL-IS resulted in a notable improvement in the transdermal permeation of aripiprazole. [Cho] [Cin] exhibited the most pronounced enhancement in permeation efficacy, as evidenced by a 14-fold increase in the cumulative permeation quantity over a 10 h period compared to the usage of IL or IS alone. Additionally, [Cho] [Ace], [Cho] [Tar], and [Cho] [Ole] did not have significant permeation enhancement effects under when IL-IS was applied. As shown in Figure 5(F), the effect of [Cho] [Cin] concentrations of 0–20% on the transdermal permeation of aripiprazole with a negative current intensity of 0.3 mA. The transdermal penetration of aripiprazole was observed to increase with the rise in the concentration of [Cho] [Cin]. The transdermal penetration of aripiprazole was within a specific concentration range. In addition, within a specific range of concentrations, there existed a linear relationship between the concentration of IL and the efficacy of drug permeation (Li *et al.* 2023).

#### 3.3.5. The effect of transdermal penetration enhancement of IL-IS on alkaline drugs

As IL-IS could inhibit the transdermal migration of acidic and neutral drugs but increase the migration of alkaline drugs, two alkaline drugs, butenafine, and terazosin were chosen for experimentation, and it was determined that IL-IS could indeed increase the transdermal penetration of alkaline drugs (Li *et al.* 2024). The impact on the transdermal permeation of weak basic drugs was confirmed based on the findings, with a [Cho] [Cin] concentration of 20% and a negative current of 0.3 mA. The results were shown in Figures 5(G,H). The permeation of butenafine applying IL-IS was found to be ∼16.5-fold/11.0-fold higher compared to its permeation applying IL/IS. Similarly, the permeation of terazosin applying IL-IS was ∼2.6-fold/5.2-fold higher compared to its permeation applying IL/IS. These results suggested that combining use of IL-IS significantly enhanced transdermal drug permeation, demonstrating its efficacy over the individual use of IL or IS alone. No significant correlation was observed between the drug retention in the skin and the concentration of ILs, types of ILs, current intensity, and other variables. The amount to which a drug was retained in the skin may mostly be attributed to the properties of the drug itself.

## 4. Study on the mechanism of transdermal penetration enhancement

### 4.1. The effects of IL-IS system on rats skin SC IR spectral signals

The characteristic peaks of ATR-FTIR are shown in Table S2. The vibration peaks seen at ∼2850 and 2920 cm^−1^ were attributed to the symmetric vibration (v) CH_2_ and asymmetric vibration (vas) of CH_2_ within the lipid chain. The presence of a vibration peak at around 1735 cm^−1^ was ascribed to the symmetric vibration (v) CH_2_ and the asymmetric vibration (vas) of CH_2_ within the lipid chain. The observation of a peak blue shift suggested a rise in the gauche/trans ratio, which in turn implied an augmentation in the fluidity of the SC lipids and a disruption of the lamellar organization within the bilayer. Following the use of IL, IS, and IL-IS technologies, it was shown that the vibration peaks in the skin, namely between 2850 and 2920 cm^−1^, experienced a discernible blue shift. This phenomenon suggested that the absorption of the drugs might potentially be enhanced by facilitating the fluidity of the SC lipid bilayer and inducing disorder in the lipid chain. Noteworthy changes were also noted in the tensile vibrations associated with the amide I band (1643 cm^−1^) and amide II band (1537 cm^−1^) of keratin. This observation indicated a modification in the secondary structure of keratin, potentially leading to a conformational shift in the keratin protein.

### 4.2. The effects of IL-IS system on rat skin intercellular space and irritation

**Figure 6.**
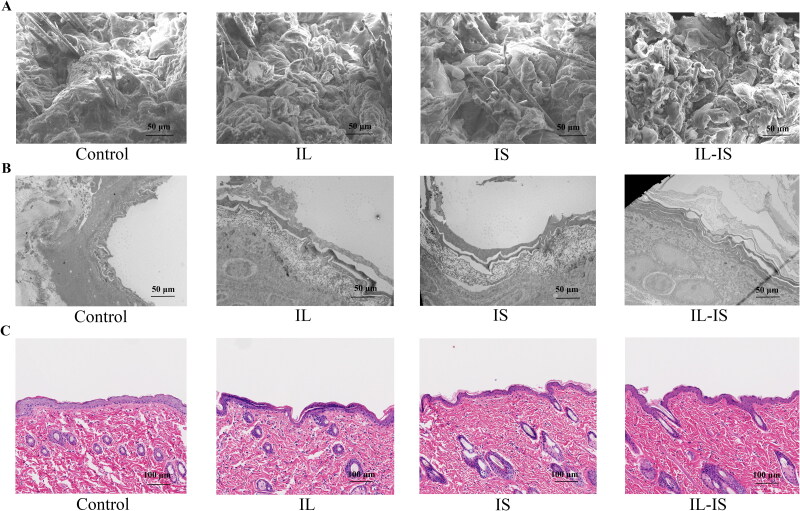
SEM, TEM images, and histopathological evaluation of skin samples for different methods treated. (A) SEM of skin applicated control gel, IL gel, IS and IL-IS for 24 h. (B) TEM of skin applicated control gel, IL gel, IS and IL-IS for 24 h. (C) Histopathological evaluation of skin applicated control gel, IL gel, IS and IL-IS for 7 days.

The results showed that there was no visible exfoliation of the SC on the skin of rats treated with control gel (Figure 6(A)). However, a noticeable exfoliation of SC in the skin was detected following treatment with IL and IS. After undergoing IL-IS treatment, a significant quantity of SC fragments was observed to be exfoliated off the surface of the skin. Hence, the employment of IL, IS, and IL-IS technologies had the potential to induce lipid dissolution between keratinocytes or diminish the integrity of tight junctions among keratinocytes. The fluidity of phospholipids in the SC was elevated, leading to the detachment and shedding of the outer layer of keratinocytes, which may be one of the reasons for the increase of penetration in skin (Qian *et al.* 2024). As shown in Figure 6(B), the skin that was treated with control gel displayed a densely arranged layer of keratinocytes and firmly packed keratin. Following the IL or IS treatment, there was a marginal increase observed in the intercellular gap between the keratinocytes. After IL-IS treatment, the intercellular space between the keratinocytes of the skin was significantly increased. This may be due to the reduction of tight junctions between cells, resulting in detachment of the SC. The results from both TEM and SEM were consistent, suggesting that the application of IL, IS, and IL-IS had discernible effect on lipids and keratin within the SC. These effects ultimately resulted in the separation and shedding of the SC, with IL-IS demonstrating a more pronounced influence. In addition, after 7 days of treatment with ILs, IS, and IL-IS technologies, potential skin irritation caused by transdermal treatments was evaluated by histopathological examination (Figure 6(C)). Upon treatment with IL, IS, and IL-IS technologies, no inflammatory cell infiltration was found in the epidermis and dermis of normal rat skin. Moreover, while comparing to the control and IL groups, it was seen that the sweat gland in the IS and IL-IS groups underwent a transformation from an oval shape to a spindle shape. This observation implied that IS might exert an influence on sweat glands. It is imperative to conduct further investigations to ascertain the long-term toxicological effects of these treatments.

### 4.3. The mechanism of transdermal of IL-IS system on rat skin

**Figure 7.**
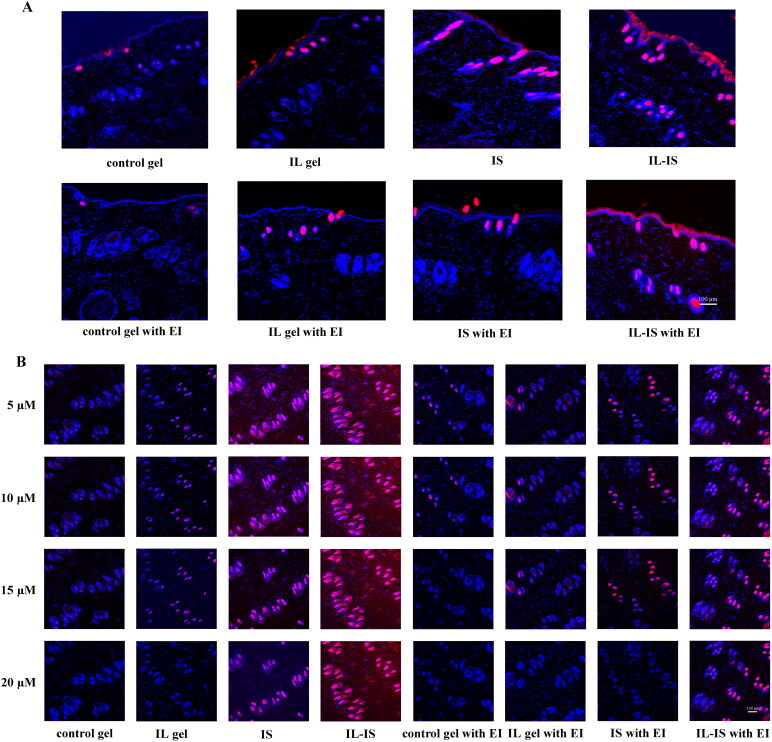
CLSM diagrams of the cross section and surface of the skin. (A) CLSM images of rhodamine B transported through skin treated by control gel, IL gel, is, IL-IS, control gel with endocytosis inhibitors, IL gel with endocytosis inhibitors, is with endocytosis inhibitors, and IL-IS with endocytosis inhibitors; (B) CLSM images of rhodamine B transported surface of the skin treated by control gel, IL gel, is, IL-IS, control gel with endocytosis inhibitors, IL gel with endocytosis inhibitors, is with endocytosis inhibitors, and IL-IS with endocytosis inhibitors.

Figure 7 displayed CLSM diagrams of the cross-section (Figure 7(A)) and surface of the skin (Figure 7(B)), respectively. The results revealed a clear decline in fluorescence intensity and depth following the application of control, IL, IS, and IL-IS. Specifically, the order of decreasing intensity and depth was observed as IL-IS > IS > IL > Control. These results suggested that the IL, IS, and IL-IS technologies had the potential to enhance the transdermal penetration of rhodamine B, with IL-IS exhibiting the most efficacy among the three approaches.

The penetration of drugs through the SC barrier typically occurs via three distinct pathways: intercellular, intracellular, and follicular (such as hair follicles, sebaceous glands, and sweat glands) pathways. This study employed DAPI as a fluorescent dye to label the cellular localization and visualize the transmission pathway of rhodamine B. As shown in Figure 7, there was an overlap in fluorescence between rhodamine B and DAPI at the precise sites of sweat glands and hair follicles. Specifically, the blue fluorescence emitted by DAPI enveloped the red fluorescence emitted by rhodamine B, indicating that the transmission of rhodamine B primarily occurred through accessory organs, such as hair follicles, sebaceous glands, and sweat glands. In addition, after the treatment with endocytosis inhibitors, the fluorescence intensity in the control group exhibited no substantial alteration, whereas it was partially suppressed in the IL, IS, and IL-IS groups. This observation implied that the IL, IS, and IL-IS technologies had the potential to enhance drug endocytosis, thereby facilitating the transdermal penetration of drugs. ILs and IS had the potential to enhance the transdermal penetration of drugs through multiple mechanisms, and the synergistic effect was better. In addition, the transdermal delivery of peptide, polysaccharide, and protein macromolecular drugs was through the pathways of skin appendages and endocytosis, which was consistent with the previously reported transdermal delivery efficacy of ILs and IS technologies for peptides and polysaccharides (Niu *et al.* 2019, Zheng *et al.* 2020, Liu T *et al.* 2024). IL-IS technology may have the potential to promote the transdermal penetration of biomacromolecular drugs.

## 5. Conclusions

In this study, the IL-IS technology was developed and evaluated for its effectiveness in facilitating transdermal drug delivery. Five groups of choline-based ILs with varying acid ligands were synthesized successfully, demonstrating significant solubilization effects on three model drugs: apremilast, aripiprazole, and indomethacin. *In vitro* penetration tests indicated that the ILs, particularly [Cho] [Cin], significantly enhanced the transdermal penetration of the model drugs. The quantity of drug permeation showed a positive linear correlation with the concentration of the ILs. Following the integration of IS, it was noted that IL-IS technology has the potential to improve the transdermal penetration of weak alkaline drugs. The permeation enhancement of [Cho] [Cin] on aripiprazole was found to be directly proportional to the intensity of the current, indicating that the rate of drug permeation can be adjusted by controlling the current intensity in the later stages. Findings from *in vivo* studies indicated that IL and/or IS technology primarily facilitate drug penetration into the skin through skin appendages, with potential involvement of endocytosis in this process. The inclusion of cinnamic acid in ionic liquids (ILs) demonstrated a notable enhancement in the transdermal permeation of three poorly soluble drugs, with aripiprazole showing potential for further improvement through the integration of IS technology. This investigation introduced a novel approach and potential for enhancing drug transdermal permeation through the synergistic use of IL-IS technology. Further studies were warranted to evaluate the safety and optimize the formulations of IL-IS technology.

## Supplementary Material

Supplemental Material

## Data Availability

The data used in this study was available by email to the corresponding author.
