# Supplementary material for: Ionic liquid-iontophoresis mediates transdermal delivery of sparingly soluble drugs
Source: Drug Deliv. 2025 Apr 21;32(1):2489730. doi: 10.1080/10717544.2025.2489730 (PMC12013143; doi:10.1080/10717544.2025.2489730)
Supplement: Supplemental Material [file IDRD_A_2489730_SM9753.docx]

Supplemental Table S1 ^1^H-NMR of IL.

| IL | δ(ppm, ^1^H-NMR) |
| --- | --- |
| [Cho] [Ace] | 4.02 – 3.79 (m, 2H), 3.68 – 3.31 (m, 3H), 3.08 (s, 9H), 1.79 (s, 3H). |
| [Cho] [Tar] | 4.20 (s, 1H), 4.00 – 3.77 (m, 2H), 3.67 – 3.51 (m, 1H), 3.51 – 3.35 (m, 2H), 3.08 (s, 9H). |
| [Cho] [Ger] | 5.54 (s, 1H), 5.11 (dd, J = 20.9, 14.4 Hz, 1H), 4.01 – 3.78 (m, 2H), 3.67 – 3.30 (m, 3H), 3.08 (s, 9H), 2.38 – 1.88 (m, 4H), 1.80 (s, 2H), 1.70 – 1.62 (m, 1H), 1.62 – 1.42 (m, 6H). |
| [Cho] [Cin] | 7.50 (d, J = 7.0 Hz, 2H), 7.37 – 7.23 (m, 4H), 6.40 (d, J = 16.1 Hz, 1H), 3.88 (dt, J = 31.2, 4.6 Hz, 2H), 3.45 – 3.33 (m, 2H), 3.05 (s, 9H). |
| [Cho] [Ole] | 5.52 – 5.02 (m, 2H), 4.04 – 3.76 (m, 2H), 3.67 – 3.31 (m, 3H), 3.08 (s, 9H), 2.02 (dd, J = 17.6, 9.7 Hz, 2H), 1.92 (d, J = 6.0 Hz, 3H), 1.31 (dd, J = 101.3, 8.6 Hz, 22H), 0.78 (q, J = 6.8 Hz, 3H). |

Supplemental Table S2 ATR-FTIR peak shifts of the SC after treatment with different group *^a^*.

|  |  | Control | IL | IS | IL+IS |
| --- | --- | --- | --- | --- | --- |
| Lipid | CH_2_, Asymm (cm^−1^) | 2918.18 | 2918.97 | 2918.62 | 2919.57 |
|  | CH_2_, Symm (cm^−1^) | 2850.08 | 2850.76 | 2850.54 | 2851.09 |
|  | C = O (cm^−1^) | 1735.42 | 1742.93 | 1742.67 | 1742.84 |
| Keratin | NH-C = O (cm^−1^) | 1643.00 | 1643.31 | 1642.94 | 1643.41 |
|  | OC-N (cm^−1^) | 1537.22 | 1537.86 | 1538.34 | 1538.92 |

*^a^* Data presented as the mean ± SD (n =3).
